# “Pericoronary adipose tissue attenuation as a marker of global coronary inflammation rather than lesion-specific risk”

**DOI:** 10.3389/fcvm.2026.1863906

**Published:** 2026-06-19

**Authors:** Ernestas Dvinelis, Silvija Sakalauske, Guste Cesnaite, Medeine Kapacinskaite, Radoslavas Stasilo, Greta Vrublevska, Justinas Bacevicius, Algridas Tamosiunas, Sigita Glaveckaite

**Affiliations:** 1 Clinic of Cardiac and Vascular Diseases, Institute of Clinical Medicine, Faculty of Medicine, Vilnius University, Vilnius, Lithuania; 2 Center of Cardiology and Angiology, Vilnius University Hospital Santaros Klinikos, Vilnius, Lithuania; 3 Department of Radiology, Nuclear Medicine and Medical Physics, Institute of Biomedical Sciences, Vilnius University, Vilnius, Lithuania

**Keywords:** atherosclerosis, coronary artery disease, major adverse cardiovascular events, pericoronary adipose tissue, vascular inflammation

## Abstract

**Aims:**

Pericoronary adipose tissue (PCAT) attenuation is a non-invasive imaging marker of coronary vascular inflammation. However, its relationship with clinical outcomes and its relevance at different levels of analysis remain incompletely understood. This study aimed to investigate the association between PCAT attenuation and major adverse cardiovascular events (MACE), with a focus on its role as a marker of coronary inflammatory burden at the lesion and patient levels.

**Methods and results:**

In this retrospective cohort study, 772 participants were included (56.6% men). Lesions from patients who experienced MACE exhibited higher (less negative) PCAT attenuation compared with those without events. PCAT attenuation was assessed at the patient and lesion levels. Associations with MACE were analyzed using logistic regression and mixed-effects models. ROC analysis evaluated predictive performance. Linear mixed-effects models showed lesion-level mean and maximum PCAT attenuation were higher in patients with MACE (ΔHU = −1.941, 95% CI −3.563 to −0.320; *p* = 0.019, and ΔHU = −2.899, 95% CI −4.838 to −0.961, *p* = 0.003, respectively), including after adjustment for cardiovascular risk factors. However, lesion-level PCAT attenuation was not associated with MACE in generalized linear mixed-effects models. At the patient level, higher PCAT attenuation values were consistently associated with increased hazard of MACE during follow-up in both univariable (HR 1.046, 95% CI 1.013–1.081; *p* = 0.006) and multivariable analyses (HR 1.014, 95% CI 1.004–1.024; *p* = 0.006). Despite these associations, discriminatory performance of PCAT attenuation was modest (AUC 0.591, 95% CI 0.534–0.644), indicating limited ability to distinguish individuals at risk.

**Conclusions:**

PCAT attenuation is associated with adverse cardiovascular outcomes at the patient level, supporting its role as a marker of global coronary inflammatory burden, with stronger, more consistent associations at the patient level than at the lesion level. While it reflects biologically relevant inflammatory processes, its limited discriminatory performance suggests that PCAT attenuation is best interpreted as a complementary marker within the broader context of clinical and imaging risk assessment.

## 1 Introduction

Vascular inflammation in coronary artery disease (CAD) plays a key role in the progression and destabilization of arterial plaques (1). Inflammation in the coronary vessel wall releases cytokines and attracts immune cells, disrupting the histological structure of the surrounding fat tissue and altering its water-lipid composition, thereby increasing pericoronary adipose tissue (PCAT) density (2).

Observational studies show that pericoronary fat attenuation is markedly higher around culprit lesions in acute coronary syndromes, reflecting more intense local inflammation (3). Since pericoronary fat attenuation changes over time with shifts in vascular inflammation, it offers a dynamic measure of disease activity rather than a static structural marker (4). However, variability in PCAT measurement limits reproducibility and clinical interpretation (5). The perivascular Fat Attenuation Index (FAI) was developed to standardize assessment and improve prognostic value (6). FAI measures the impact of coronary inflammation on perivascular adipose tissue and provides insights into disease activity beyond anatomical imaging (7). As a result, elevated FAI values can serve as a marker of coronary risk, identifying patients with increased risk of adverse cardiovascular outcomes and plaque vulnerability (1). The predictive value of FAI has been confirmed in diverse cohorts, including patients with type 2 diabetes, supporting its role in risk stratification (8). In addition to its diagnostic value, FAI can serve as a biomarker of treatment response, with early data showing that a reduction in pericoronary fat attenuation corresponds to lipid-lowering treatment efficacy (9). PCAT measurements around coronary stents have also shown potential in predicting in-stent restenosis (10). In summary, PCAT attenuation evaluation is an important step forward in non-invasive cardiovascular imaging, linking anatomical assessment with underlying vascular inflammation, and can serve as a valuable tool for risk re-evaluation in primary and secondary prevention.

The aim of this study was to assess the relationship and predictive value of PCAT attenuation for major adverse cardiovascular events (MACE) using both lesion-level and patient-level analyses in patients with coronary atherosclerosis, enabling direct comparison of their relative clinical relevance. By combining association and predictive analyses, this study offers new insights into the role of PCAT attenuation in cardiovascular risk, emphasizing its value as a marker of global coronary inflammation rather than lesion-specific risk.

## 2 Materials and methods

### 2.1 Study population

A retrospective cohort study was performed at Vilnius University Hospital Santaros Klinikos's Cardiology and Angiology Center. The study protocol was approved by the Vilnius Regional Biomedical Research Ethics Committee (2023/9-1538-994).

**Figure 1:**
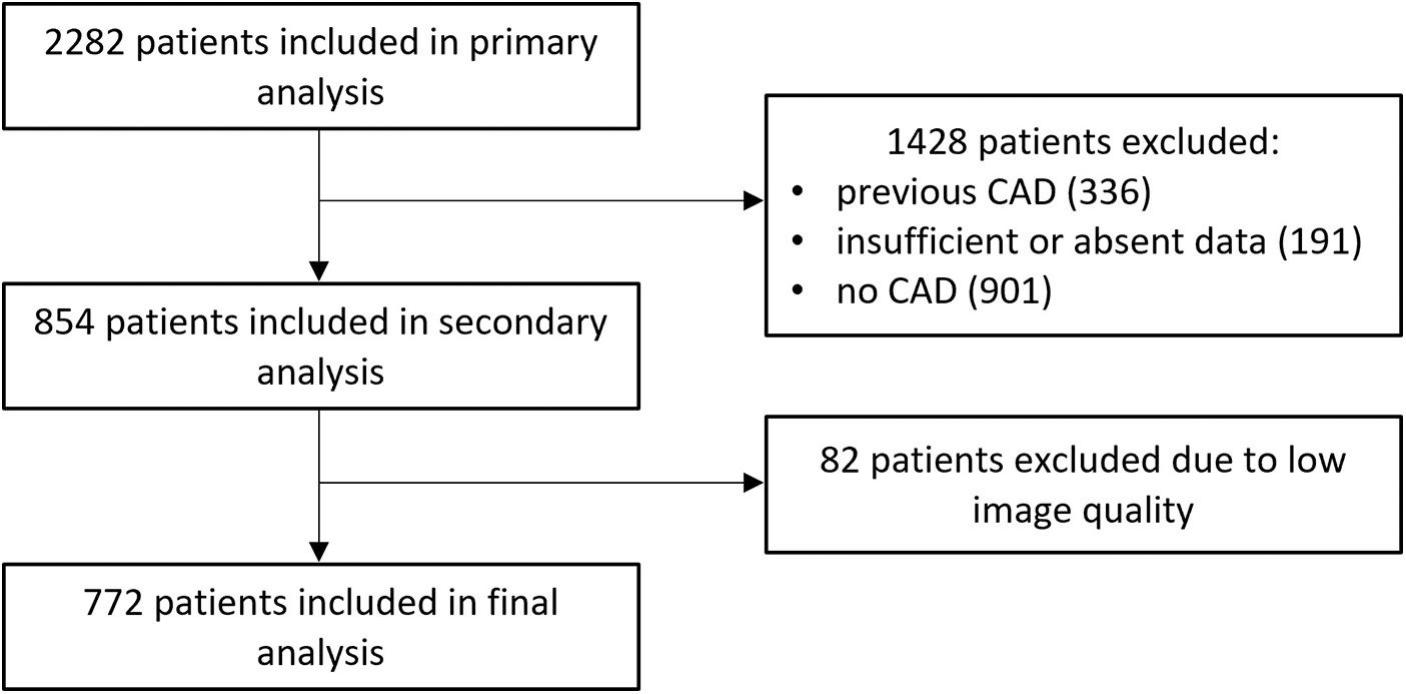
Flow chart of the study. CAD, coronary artery disease.

An analysis was conducted on stable, symptomatic patients who had coronary computed tomography angiography (CTA) for suspected CAD between January 2018 and December 2019. Patients with at least one coronary plaque in any vessel and a vessel diameter exceeding 2 mm were classified as having coronary atherosclerosis and included in the analysis. Participants were excluded if they had existing CAD, such as a history of myocardial infarction (MI), percutaneous coronary intervention (PCI), or surgical revascularisation. Additionally, cases with poor coronary CTA image quality—such as those affected by motion artifacts, insufficient contrast enhancement, or incomplete coronary artery visualization—were excluded from the analysis. Figure 1 presents a detailed flow chart.

Data, including demographic details, clinical characteristics, cardiovascular risk factors [such as smoking, diabetes, hypertension, dyslipidemia, obesity (BMI), and family history], and symptoms, were gathered from electronic health records at the time of referral for coronary CTA. Outcomes were specified as MACE, which includes acute myocardial infarction, stroke, cardiovascular mortality, and coronary revascularisation. Outcomes were evaluated at a specific point in time by searching electronic health records for relevant International Classification of Diseases codes.

### 2.2 Coronary CTA image acquisition and pericoronary adipose tissue analysis

ECG-triggered coronary CTA images were obtained using a Revolution 256-row detector computed tomography (CT) system (GE Healthcare). The scan covered 160 mm axially, with a slice thickness of 0.625 mm and a gantry rotation time of 280 ms. The tube voltage was adjusted according to the patient's body size, between 100 and 120 kV. PCAT attenuation values were corrected for tube voltage and divided by a conversion factor of 1.11485 if the coronary CTA scan was performed at a tube voltage of 100 kV, as previously described (11, 12).

Metoprolol was administered orally or intravenously to achieve a target heart rate of <65 beats per minute. Data acquisition was initiated using a contrast tracking technique. A nonionic iodine contrast agent, iopromide (Ultravist 370 mg I/mL), was injected intravenously at 5 mL/sec, with a total volume of 60–80 mL depending on body mass, followed by a saline flush.

Pericoronary adipose tissue (PCAT) attenuation was assessed on coronary CT angiography using semi-automated software (QAngio CT RE 3.2.14.4, Medis Medical Imaging Systems, the Netherlands). For each vessel, a three-dimensional perivascular region of interest was generated automatically, defined as a radial distance from the outer vessel wall equal to the local vessel diameter (12). To avoid the effects of the aortic wall, we excluded the proximal ∼10 mm of the coronary arteries. Within this region, adipose tissue was identified using a predefined attenuation threshold of −190 to −30 Hounsfield Units (HU), consistent with previously validated definitions of adipose tissue in coronary CT studies (13, 14). The mean attenuation of all voxels within this adipose tissue range was calculated and recorded as mean PCAT attenuation. Additionally, the minimum and maximum attenuation values within the same region were also obtained. All PCAT attenuation measurements were performed automatically. This measurement approach is consistent with the methodology used for the FAI, reflecting the average attenuation of perivascular adipose tissue as a surrogate marker of coronary inflammation. As part of quality control, analyses were restricted to coronary vessels ≥2 mm in diameter, attenuation thresholds were standardized and tube-voltage correction was applied, and PCAT measurements were performed using a standardized automated workflow to minimise operator-dependent variability. In addition, studies with inadequate image quality were excluded prior to analysis, as noted above.

### 2.3 Statistical analysis

All statistical analyses were conducted using SPSS (version 31.0) and Microsoft Excel software. Data were analyzed using frequency tables. To test data normality, the Kolmogorov–Smirnov and Shapiro–Wilk tests were used. Continuous variables are presented as mean ± standard deviation, and categorical variables as counts and percentages. Group comparisons between patients with and without major adverse cardiovascular events (MACE) were performed using independent-samples *t*-tests or Mann–Whitney *U*-tests for continuous variables and *χ*^2^ or Fisher's exact tests for categorical variables, as appropriate.

Patient-level PCAT attenuation metrics were derived by aggregating segment-level values to obtain mean, minimum, and maximum values per patient. Associations between patient-level PCAT attenuation and MACE during follow-up were assessed using univariable and multivariable Cox proportional hazards regression models, with multivariable analyses adjusted for age, gender, hypertension, diabetes mellitus, dyslipidemia, smoking status, obesity (BMI), family history of coronary artery disease, and CAD stenosis severity, and plaque burden. Lesion-level PCAT attenuation was quantified by calculating the mean, minimum, and maximum attenuation values from the perivascular adipose tissue surrounding each identified coronary lesion. Lesion-level analyses were performed using linear mixed-effects models to evaluate differences in PCAT attenuation between patients with and without MACE. To account for withing-patient clustering of multiple lesions and avoid treating lesion-level observartions as fully independent, patient identifier was included as a random intercept. Both unadjusted and adjusted models were constructed, with adjustment for the cardiovascular risk factors. Variables included in multivariable analyses were selected based on established cardiovascular risk factors, clinical relevance, and previously reported associations with cardiovascular outcomes and coronary inflammation. In addition, generalized linear mixed-effects logistic regression models were used to assess the association between lesion-level PCAT attenuation and MACE. Results are presented as hazard ratios (HR) and odds ratios (OR) with 95% confidence intervals (CI).

Receiver operating characteristic (ROC) curve analysis was performed to evaluate the ability of patient-level PCAT attenuation parameters to predict MACE. The area under the curve (AUC) with 95% confidence intervals was calculated, and optimal cut-off values were determined using the Youden index (sensitivity + specificity − 1). Sensitivity and specificity at the optimal thresholds were reported. Comparisons between AUCs were performed using the DeLong test for correlated ROC curves. A *p*-value of <0.05 was considered statistically significant.

## 3 Results

### 3.1 Demographic Data

Our study involved 2,282 patients, of whom 772 (34%) were included in the analysis; 437 (56.6%) were men and 335 (43.4%) were women. Figure 1 presents the flow chart of this study. Most of the study population was aged 60 years or older, comprising 61.5% (475 individuals). A total of 7,577 segments were assessed, averaging about 9.8 segments per patient. Moderate to severe coronary lesions (stenosis of ≥50%) were found in 30.8% of the patients*.* The most common risk factors were dyslipidemia and hypertension, with 84% of the patients presenting with two or more, and 32% with three or more risk factors.

### 3.2 Pericoronary adipose tissue attenuation in relation to gender

PCAT attenuation was evaluated at the patient, vessel, and lesion levels, with analyses stratified by gender. At the patient level, mean PCAT attenuation was significantly higher in men than in women (−78.40 ± 9.61 vs. −80.42 ± 9.96 HU, *p* < 0.001); the same trend was observed at the lesion level (−76.33 ± 10.57 vs. −78.55 ± 11.31 HU, *p* < 0.001). Vessel-level analyses showed higher PCAT attenuation values in men than in women across all coronary arteries. Differences were most pronounced in the RCA (−78.11 ± 9.69 vs. −81.18 ± 10.35, *p* < 0.001). Lesion-level PCAT attenuation was significantly higher than patient-level PCAT attenuation (−77.18 ± 10.91 vs. −79.65 ± 9.60, *p* < 0.001). In addition, minimum and maximum PCAT attenuation values were consistently higher in men at both lesion and patient levels.

Men were younger than women (59.6 ± 9.9 vs. 65.2 ± 8.7 years, *p* < 0.001) and had a higher prevalence of smoking (21.5% vs. 7.5%, *p* < 0.001). The prevalence of diabetes mellitus, family history of cardiovascular disease, hypertension, and dyslipidemia was similar between groups, with no statistically significant differences observed. Table 1 shows the baseline characteristics of the study population by gender.

**Table 1:** Baseline characteristics of the study patients by gender.

Characteristics	Men	Women	*p*-value
*N*, %	437 (56.6)	335 (43.4)	
Age, ±SD (yrs)	59.6 (9.9)	65.2 (8.7)	<0.001
BMI, ±SD (kg/m^2^)	29 (4.8)	28.8 (4.6)	0.657
Smoker, %	96 (21.5)	25 (7.5)	<0.001
Diabetes, %	58 (13.3)	56 (16.7)	0.181
Family history, %	73 (16.7)	66 (19.7)	0.283
Hypertension, %	384 (87.9)	292 (87.2)	0.768
Dyslipidaemia, %	384 (87.9)	301 (89.9)	0.389
Mean lesion PCAT attenuation (HU)	−76.33 (10.57)	−78.55 (11.31)	<0.001
Minimum lesion PCAT attenuation (HU)	−88.44 (11.41)	−89.77 (12.24)	0.013
Maximum lesion PCAT attenuation (HU)	−66.99 (12.17)	−69.79 (12.12)	0.005
Mean patient PCAT attenuation (HU)	−78.40 (9.61)	−80.42 (9.96)	<0.001
Minimum patient PCAT attenuation (HU)	−97.48 (11.31)	−100.41 (11.91)	<0.001
Maximum patient PCAT attenuation (HU)	−58.09 (12.94)	−59.08 (13.13)	0.001
Mean RCA PCAT attenuation (HU)	−78.11 (9.69)	−81.18 (10.35)	<0.001
Mean LAD PCAT attenuation (HU)	−80.14 (9.05)	−81.08 (9.81)	0.006
Mean LCx PCAT attenuation (HU)	−75.86 (9.41)	−78.12 (9.27)	<0.001

PCAT, pericoronary adipose tissue; HU, Hounsfield units; BMI, body mass index; *N*, number of patients; SD, standard deviation; RCA, right coronary artery; LAD, left anterior descending artery; LCx, left circumflex artery.

### 3.3 Pericoronary adipose tissue attenuation, cardiovascular risk factors, and clinical outcomes

#### 3.3.1 Clinical characteristics and cardiovascular risk factors according to MACE status

PCAT attenuation was evaluated at both the patient and lesion levels, and analyses were stratified according to MACE status. Patients who experienced MACE were older (63.8 ± 9.8 vs. 61.8 ± 9.8 years, *p* = 0.041) and had a higher body mass index (30.6 ± 6.8 vs. 28.7 ± 4.3 kg/m^2^, *p* < 0.001) compared with those without MACE.

Smoking and diabetes were significantly more prevalent among patients with MACE (smoking: 28.8% vs. 13.3%, *p* < 0.001; diabetes: 27.0% vs. 12.7%, *p* < 0.001). Hypertension was also more frequent in the MACE group (94.6% vs. 86.2%, *p* = 0.014). The prevalence of dyslipidemia was numerically higher in patients with MACE but did not reach statistical significance (93.7% vs. 87.9%, *p* = 0.074). Family history of cardiovascular disease did not differ between groups (18.0% vs. 17.8%, *p* = 0.966). The detailed results are presented in Table 2.

**Table 2:** Baseline clinical characteristics and PCAT attenuation according to MACE status.

Characteristics	No MACE	MACE	*p*-value
*N*, %	661 (56.6)	111 (43.4)	
Age, ±SD (yrs)	61.8. (9.8)	63.8 (9.8)	0.041
BMI, ±SD (kg/m^2^)	28.7 (4.3)	30.6 (6.8)	<0.001
Smoker, %	88 (13.3)	32 (28.8)	<0.001
Diabetes, %	84 (12.7)	30 (27.0)	<0.001
Family history, %	118 (17.8)	20 (18.0)	0.966
Hypertension, %	570 (86.2)	105 (94.6)	0.014
Dyslipidaemia, %	581 (87.9)	104 (93.7)	0.074
Plaque burden mean, ±SD (%)	34.7 (16.0)	43.2 (19.4)	<0.001
Moderate to severe stenosis, %	150 (22.7)	88 (79.3)	<0.001
Mean lesion PCAT attenuation (HU)	−77.96 (9.16)	−75.82 (7.8)	0.010
Minimum lesion PCAT attenuation (HU)	−89.04 (9.94)	−88.51 (8.05)	0.532
Maximum lesion PCAT attenuation (HU)	−68.75 (9.93)	−65.97 (8.50)	0.006
Mean patient PCAT attenuation (HU)	−79.54 (5.64)	−77.82 (5.17)	0.002
Minimum patient PCAT attenuation (HU)	−99.05 (5.91)	−97.35 (5.30)	0.003
Maximum patient PCAT attenuation (HU)	−58.76 (6.24)	−57.03 (6.14)	0.007

*P*CAT, pericoronary adipose tissue; HU, Hounsfield units; BMI, body mass index; *N*, number of patients; SD, standard deviation.

Over a median follow-up of 70 months (about 5.8 years), 111 patients (14.4%) experienced MACE, with an annual rate of 2.5%. Of these, 53 (48%) were elective coronary revascularisations, 40 (36%) involved non-fatal MI or unstable angina, 11 (10%) were strokes, and 7 (6%) led to cardiovascular deaths. Among MACE cases, 69 (62%) were in men, and 42 (38%) in women.

#### 3.3.2 Lesion-level analyses

In unadjusted linear mixed-effects models accounting for clustering of lesions within patients, lesions from patients who experienced MACE demonstrated significantly higher (less negative) mean PCAT attenuation compared with lesions from patients without MACE (ΔHU = −1.941, 95% CI −3.563 to −0.320; *p* = 0.019). Similarly, lesion-level maximum PCAT attenuation was significantly higher among patients with MACE (ΔHU = −2.899, 95% CI −4.838 to −0.961; *p* = 0.003). In contrast, lesion-level minimum PCAT attenuation did not differ between groups (*p* = 0.662).

After adjustment for age, gender, hypertension, diabetes mellitus, dyslipidemia, smoking status, obesity (BMI), and family history of coronary artery disease, the association between lesion-level mean PCAT attenuation remained significantly higher in patients who experienced MACE (adjusted ΔHU = −2.190, 95% CI −3.883 to −0.498; *p* = 0.011). Maximum lesion-level PCAT attenuation also remained significant after adjustment (adjusted ΔHU = −3.283, 95% CI −5.315 to −1.251; *p* = 0.002).

When lesion-level PCAT attenuation was evaluated using generalized linear mixed-effects logistic regression models with MACE as the outcome, none of the lesion-level PCAT attenuation metrics were significantly associated with MACE (mean PCAT attenuation OR 0.987, 95% CI 0.968–1.007; *p* = 0.200; minimum PCAT attenuation OR 0.998, 95% CI 0.980–1.016; *p* = 0.791; maximum PCAT attenuation OR 0.985, 95% CI 0.966–1.005; *p* = 0.143). Results of lesion-level mixed-effects analyses are shown in Table 3.

**Table 3:** Lesion-level mixed-effects analysis of PCAT attenuation and MACE.

A. Linear mixed-effects models (Outcome: PCAT attenuation)				
Predictor	Unadjusted *Δ*HU (95% CI)	*p*-value	Adjusted *Δ*HU (95% CI)*	*p*-value
Mean lesion PCAT attenuation	−1.941 (−3.563 to −0.320)	0.019	−2.190 (−3.883–0.498)	0.011
Minimum lesion PCAT attenuation	−0.387 (−2.128–1.353)	0.662	0.918 (−2.739–0.903)	0.322
Maximum patient PCAT attenuation	−2.899 (−4.838 to −0.961)	0.003	−3.283 (−5.315 to −1.251)	0.002

B. Logistic mixed-effects models (Outcome: MACE)		
**Predictor**	**Odds Ratio (95% CI)**	***p*-value**
Mean lesion PCAT attenuation	0.987 (0.968–1.007)	0.200
Minimum lesion PCAT attenuation	0.998 (0.980–1.016)	0.791
Maximum patient PCAT attenuation	0.985 (0.966–1.005)	0.143

PCAT, pericoronary adipose tissue; HU, Hounsfield units; MACE, major cardiovascular adverse events; CI, confidence interval.

#### 3.3.3 Patient-level analyses

In univariable analyses, higher patient-level PCAT attenuation values were significantly associated with increased hazard of MACE during follow-up. Mean PCAT attenuation demonstrated a significant association with MACE (HR 1.046 per 1 HU increase, 95% CI 1.013–1.081; *p* = 0.006), as did minimum PCAT attenuation (HR 1.041, 95% CI 1.010–1.074; *p* = 0.010) and maximum PCAT attenuation (HR 1.039, 95% CI 1.009–1.071; *p* = 0.011).

These associations remained significant after adjustment for age, gender, hypertension, diabetes mellitus, dyslipidemia, smoking status, obesity (BMI), family history of coronary artery disease, CAD stenosis severity, and plaque burden. Adjusted HRs were 1.014 (95% CI 1.004–1.024; *p* = 0.006) for mean PCAT attenuation and 1.010 (95% CI 1.003–1.018; *p* = 0.009) for maximum PCAT attenuation, whereas minimum PCAT attenuation was no longer significantly associated with MACE after adjustment (HR 1.006 (95% CI 0.997–1.014; *p* = 0.199). Patient-level Cox proportional hazards analyses are presented in Table 4.

**Table 4:** Association between patient-level PCAT attenuation and MACE during follow-up.

Predictor	Univariable HR (95% CI)	*p*-value	Multivariable HR (95% CI)	*p*-value
Mean patient PCAT attenuation	1.046 (1.013–1.081)	0.006	1.014 (1.004–1.024)	0.006
Minimum patient PCAT attenuation	1.041 (1.010–1.074)	0.010	1.006 (0.997– 1.014)	0.199
Maximum patient PCAT attenuation	1.039 (1.009–1.071)	0.011	1.010 (1.003–1.018)	0.009

PCAT, pericoronary adipose tissue; MACE, major cardiovascular adverse events; CI, confidence interval; OR, odds ratio.

#### 3.3.4 Predictive performance

When lesion-level PCAT attenuation was assessed using generalized linear mixed-effects logistic regression models with MACE as the outcome, none of the lesion-level PCAT attenuation metrics showed a significant association with MACE. Since MACE is a patient-level outcome, and lesion-level PCAT attenuation was not independently associated with MACE, predictive analyses were conducted at the patient level.

**Figure 2:**
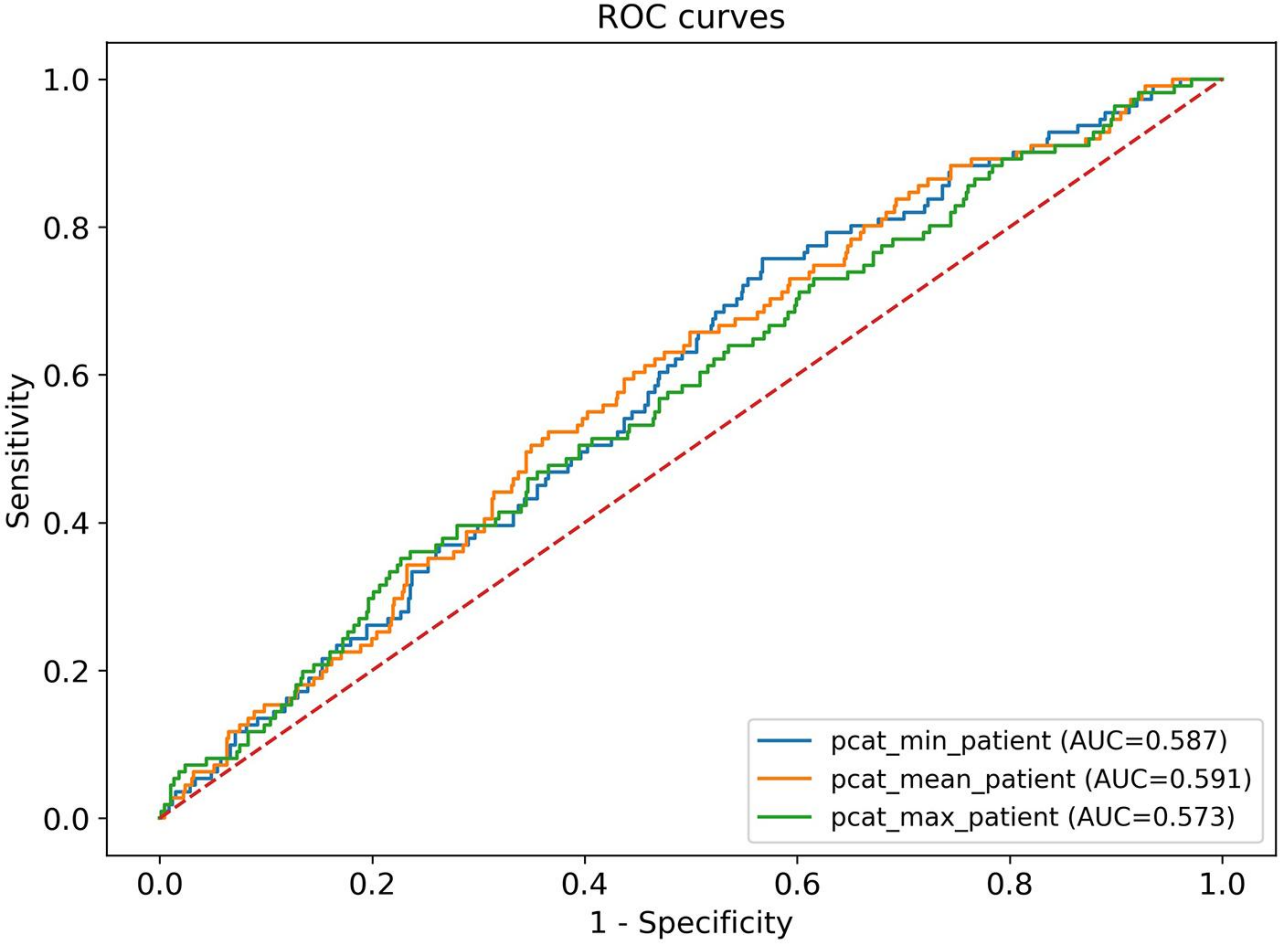
ROC curves of patient-level PCAT parameters for predicting MACE. PCAT, pericoronary adipose tissue; MACE, major cardiovascular adverse 3 events; ROC curves, receiver operating characteristic curves; AUC, area under the curve.

ROC analysis demonstrated limited discriminatory performance of patient-level PCAT parameters for predicting MACE. Mean PCAT showed the highest AUC (0.591, 95% CI: 0.534–0.644), followed by minimum PCAT (AUC 0.587, 95% CI: 0.531–0.638) and maximum PCAT (AUC 0.573, 95% CI: 0.516–0.629). The optimal cut-off value for mean PCAT was −79.7 HU, yielding a sensitivity of 65.8% and specificity of 50.1%. Maximum PCAT showed higher specificity (77.3%) but lower sensitivity (35.1%) at a cut-off of −54.2 HU. Minimum PCAT demonstrated higher sensitivity (75.7%) but lower specificity (43.3%) at a threshold of −100.4 HU. Pairwise comparison using the DeLong test revealed no statistically significant differences between the evaluated PCAT metrics (all *p* > 0.05). The results are presented in Table 5 and Figure 2.

**Table 5:** Diagnostic performance of patient-level PCAT attenuation parameters (mean across arteries) for predicting MACE.

Variable	AUC (95% CI)	Optimal cut-off (HU)	Sensitivity (%)	Specificity (%)
Mean patient PCAT attenuation	0.591 (0.534–0.644)	−79.7	65.8	50.1
Minimum patient PCAT attenuation	0.587 (0.531–0.638)	−100.4	75.7	43.3
Maximum patient PCAT attenuation	0.573 (0.516–0.629)	−54.2	35.1	77.3

PCAT, pericoronary adipose tissue; HU, Hounsfield units; MACE, major cardiovascular adverse events; CI, confidence interval.

## 4 Discussion

In this retrospective cohort study, we examined the relationship between pericoronary adipose tissue (PCAT) attenuation and major adverse cardiovascular events (MACE) at both lesion and patient levels. PCAT attenuation values were consistently higher in men than in women across patient, vessel, and lesion analyses. Lesions from patients who experienced MACE showed higher PCAT attenuation; however, after accounting for clustering within patients, lesion-level PCAT attenuation was not independently associated with outcomes. In contrast, patient-level PCAT attenuation demonstrated consistent independent associations with MACE. Despite these associations, discriminatory performance was modest (AUC < 0.60), suggesting that PCAT attenuation primarily reflects global coronary inflammatory burden rather than lesion-specific risk and has limited value as a standalone predictive marker.

### 4.1 PCAT attenuation and its association with MACE

In this study, higher PCAT attenuation was observed in patients who experienced MACE, supporting its association with adverse cardiovascular outcomes. These findings support the idea that PCAT attenuation reflects underlying coronary inflammation associated with cardiovascular risk, as PCAT attenuation on coronary CTA is considered an imaging surrogate of coronary inflammation, with less negative attenuation values indicating inflammation-related changes in adipose tissue (1, 15).

Our results broadly align with the studies mentioned, indicating that higher perivascular or pericoronary fat attenuation is associated with a worse cardiovascular prognosis. In the CRISP-CT study, perivascular FAI provided prognostic information for cardiac mortality beyond traditional risk assessment, and subsequent analyses demonstrated that FAI further stratified risk associated with high-risk plaque features (1, 16). More recently, long-term outcome data have also supported the prognostic significance of PCAT attenuation derived from coronary CTA, although its additional predictive value appears to vary by clinical setting and the underlying burden of coronary disease (17).

In the present study, the composite MACE endpoint included elective coronary revascularisation, as it may reflect clinically relevant progression of coronary artery disease and an increased underlying inflammatory burden. In our cohort, the association between PCAT attenuation and MACE remained clear, emphasizing that PCAT reflects biologically relevant processes involved in the progression of atherosclerotic disease. However, the strength and consistency of this association varied depending on the level of analysis (lesion- vs. patient-level).

### 4.2 Lesion-level vs. patient-level PCAT attenuation

This study finds that the significance of PCAT attenuation varies across analysis levels. Lesion-level PCAT was higher in patients with MACE, but it wasn't independently associated with MACE after accounting for multiple lesions from the same patient. This reflects data hierarchy and shows the limited predictive value of individual lesion measurements. Notably, mean and maximum lesion-level PCAT attenuation remained associated with MACE after adjustment, suggesting that focal areas of increased perivascular inflammation may contribute to the overall inflammatory burden.

In contrast, patient-level mean and maximum PCAT attenuation showed consistent and independent associations with MACE. The loss of significance for minimum PCAT attenuation after adjustment may be biologically plausible, as lower attenuation values are less likely to reflect active inflammatory changes. Together, these findings suggest that PCAT attenuation may reflect overall coronary inflammation burden more consistently than lesion-specific processes. Experimental and translational studies have demonstrated that perivascular adipose tissue is biologically active and responds to inflammatory signals from the vascular wall, leading to observable changes in computed tomography attenuation (12, 18). Several imaging studies have further shown that increased PCAT attenuation is associated with high-risk plaque features and markers of plaque vulnerability, including positive remodeling, low-attenuation plaque, and necrotic core (19, 20).

From a prognostic perspective, multiple studies have shown that increased PCAT attenuation is associated with adverse cardiovascular outcomes and provides additional prognostic value beyond traditional risk factors and coronary plaque burden (1, 21). However, most previous studies have assessed PCAT at a single level of analysis, usually at the patient or vessel level.

Importantly, our study provides insights into lesion-level and patient-level PCAT attenuation. Our findings suggest that while perivascular inflammation is increased in patients with MACE, its predictive value is more robust when assessed at the patient level, reflecting the cumulative inflammatory burden of the coronary vasculature rather than focal lesion-specific processes. This is consistent with emerging evidence that systemic inflammatory activity plays a central role in cardiovascular risk, beyond the characteristics of individual plaques (22). These findings indicate that lesion-level PCAT attenuation alone may have limited value for predicting clinical outcomes.

Taken together, these findings suggest that patient-level PCAT attenuation may be more relevant to cardiovascular risk assessment than lesion-level measurements within the present study design.

### 4.3 Predictive performance of PCAT attenuation

Despite significant associations between patient-level PCAT attenuation and MACE in regression analyses, its discriminatory performance in our cohort was modest, with AUC values below 0.60 across all metrics. This suggests that although PCAT attenuation reflects biologically relevant coronary inflammatory activity, its ability to identify individual patients at risk is limited when used alone. Similar findings have been reported in previous studies, where PCAT attenuation was associated with adverse cardiovascular outcomes but demonstrated only modest standalone predictive performance, particularly when compared with or combined with established clinical and plaque-based risk markers (23, 24).

This may be explained by the fact that PCAT attenuation reflects vascular inflammation, which is more closely associated with plaque vulnerability than with clinical events alone. Indeed, previous studies have demonstrated that higher PCAT attenuation is associated with high-risk plaque characteristics (19, 25). Consistent with this, recent systematic evidence confirms that PCAT attenuation relates to both MACE risk and high-risk plaque characteristics, although considerable heterogeneity exists, probably due to differences in coronary vessel assessment and measurement techniques (5). This variability may partly explain the modest predictive performance observed in the present study.

Compared with established imaging markers such as plaque burden and stenosis severity, PCAT attenuation may provide complementary information by reflecting inflammatory activity rather than structural atherosclerotic burden alone. While positron emission tomography-based imaging offers a more direct assessment of vascular inflammation, PCAT attenuation can be derived from routine coronary CTA and may improve cardiovascular risk assessment when integrated with clinical and imaging markers as part of a multi-parametric approach (24). In addition, because PCAT attenuation reflects dynamic inflammatory processes, it may also have potential utility for monitoring treatment response and guiding preventive or targeted therapeutic strategies in future prospective studies (26).

### 4.4 Gender-related differences in PCAT attenuation

PCAT attenuation values were consistently higher in men than in women across analyses at the patient, vessel, and lesion levels. These findings indicate that PCAT attenuation may be influenced by gender-related differences in coronary inflammation and perivascular adipose tissue biology.

Multiple mechanisms may explain these differences. Experimental data suggest that sex steroids, especially estrogen, influence fat tissue by affecting preadipocyte proliferation; this may explain differences in perivascular adipose tissue between men and women, with men exhibiting a more pro-inflammatory phenotype (27, 28). Additionally, gender-related differences in atherosclerotic plaque features, coronary inflammation, microvascular, and endothelial dysfunction have been reported, which may explain higher PCAT attenuation values in men (29, 30).

In our cohort, the higher prevalence of smoking among men might have further contributed to increased PCAT attenuation, given the well-established association between smoking and vascular inflammation (31). However, the persistence of differences across various levels of analysis indicates that underlying biological factors, beyond traditional risk factors alone, are probably involved.

Overall, these findings are consistent with emerging evidence that gender differences affect coronary inflammation and imaging biomarkers, underscoring the need to consider gender-specific factors in interpreting PCAT attenuation.

## 5 Limitations and strengths

Several limitations of this study should be acknowledged. First, the single-center, retrospective observational design limits the ability to generalize findings and establish causality, and may be influenced by unmeasured or incomplete confounding factors. Although multivariable adjustment was used, some confounders may still remain. Second, mixed-effects modeling accounted for lesion clustering, but lesion-level analyses could still be affected by lesion characteristics. Also, PCAT attenuation measurements obtained from coronary CT angiography may still be affected by technical factors during image acquisition and analysis, despite efforts to standardize and correct for acquisition parameters. Information on statin therapy was excluded because of incomplete data and low pre-scan compliance, although most patients initiated treatment after coronary CTA. The effect of statins on PCAT attenuation needs more research. The composite MACE endpoint included elective coronary revascularisation, which may be influenced not only by biological disease progression but also by physician decision-making, and baseline CTA findings, potentially reducing specificity for spontaneous adverse cardiovascular events. Finally, the limited number of events, especially at the lesion level, may have affected estimate accuracy.

Despite these limitations, this study has several important strengths. It includes a relatively large cohort of patients with detailed clinical and imaging data and long-term follow-up. A key strength is the examination of PCAT attenuation across various levels of analysis, including lesion- and patient-level assessments, offering a more detailed understanding of its role in cardiovascular risk. Furthermore, the use of mixed-effects modeling enabled appropriate handling of clustered data, and the combined evaluation of association and predictive performance enhances the clinical significance of the findings. Importantly, PCAT attenuation was not assessed during the acute phase of coronary syndromes. As PCAT reflects dynamic inflammatory activity, measurements during acute events may differ, whereas our analysis aimed to capture baseline inflammation prior to clinical outcomes. Overall, these methodological approaches support a more robust and comprehensive assessment of PCAT attenuation as an indicator of coronary inflammation.

## 6 Conclusion

In this study, higher coronary PCAT attenuation was independently associated with major adverse cardiovascular events at the patient level, but not at the lesion level, after accounting for clustering. These findings suggest that patient-level PCAT attenuation may reflect global coronary inflammatory burden more consistently than lesion-level measurements. Despite this association, its discriminatory performance was modest, indicating limited value for identifying individual patients when used alone. Therefore, PCAT attenuation is best interpreted as a complementary marker alongside clinical risk factors, rather than a standalone predictive tool.

## Data Availability

The raw data supporting the conclusions of this article will be made available by the authors, without undue reservation.
